# Suicide assistance in Germany: A protocol for a multi-perspective qualitative study to explore the current practice

**DOI:** 10.1371/journal.pone.0311880

**Published:** 2024-12-13

**Authors:** Sven Schwabe, Franziska A. Herbst, Stephanie Stiel, Nils Schneider

**Affiliations:** Institute for General Practice and Palliative Care, Hannover Medical School, Hannover, Germany; PLOS: Public Library of Science, UNITED KINGDOM OF GREAT BRITAIN AND NORTHERN IRELAND

## Abstract

**Background:**

Suicide assistance is as a complex process comprising a series of stages, ranging from initial consultations with patients about end-of-life options to counselling bereaved family members. The role of healthcare professionals and other practitioners in this process varies according to national regulations and procedural requirements. After a decision of the German Federal Constitutional Court in 2020, suicide assistance is unregulated in Germany but official data about the implementation of suicide assistance is lacking.

**Aims:**

The project “ASEP–Assisted suicide in Germany: Exploring the practice” aims to generate and disseminate scientific knowledge on the practice of assisted suicide in Germany, based on the experiences of practitioners and family members.

**Methods:**

ASEP is a prospective, observational, qualitative study comprising three phases. In Phase 1, expert interviews will be conducted to identify involved professional groups, issues and stages of the implementation of suicide assistance in Germany. In phase 2, experiences of practitioners and family members, who were involved in the practice of suicide assistance in Germany, will be collected via semi-structured interviews and analysed. In phase 3, findings of phase 2 will be discussed in focus groups with experts to identify linkages between the practice of assisted suicide and the healthcare system and to develop recommendations on how these linkages should be organised.

**Discussion:**

The results of this study will gain insights into the practice of suicide assistance for the first time in Germany. The findings are expected to inform scientific societies, professional association, and policy makers about the current practice and provide recommendations for better linkages of assisted suicide with the healthcare system. In this way, the project provides an evidence-based impetus for a more transparent and well-founded handling of assisted suicide in the German healthcare system.

**Trial registration:**

The study was prospectively registered in the German Clinical Trials Register (Deutsches Register Klinischer Studien) (Registration N° DRKS00034574; date of registration: 04 July 2024). The study is searchable under the International Clinical Trials Registry Platform Search Portal of the World Health Organization, under the German Clinical Trials Register number.

## Introduction

Assisted dying practices are discussed controversially in the fields of ethics, medicine, and politics. Across the world, euthanasia or physician assisted dying (PAS) are legal in 19 jurisdictions: The Netherlands, Belgium, Luxemburg, Colombia, Canada, and two Australian states permit euthanasia; Switzerland, Austria and ten US jurisdictions permit only PAS [1, 2]. In all of these jurisdictions, there are substantive and procedural requirements and safeguards in place to regulate access to and the practice of assisted dying [3]. In some countries, there are guidelines for healthcare professionals, who are involved in suicide assistance. These guidelines delineate for example the tasks and responsibilities of the professionals, medications, communicational and legal aspects.

The implementation of medical assistance in dying is described as a complex process comprising a series of stages, ranging from initial consultations with patients about end-of-life options to counselling bereaved family members after the event [4]. According to a scoping review about the implementation of medical assistance in dying, the process was reported to unfold over seven steps: 1) communication and counselling, 2) assessment of eligibility and regulatory oversight, 3) prescription of lethal drugs, 4) death planning, 5) supervision of the suicide (and management of any complications), 6) death reporting, and 7) provision of aftercare for bereaved family members [4, 5]. The role of healthcare professionals and other practitioners in these procedures varies according to national regulations and procedural requirements. Across all countries, physicians play a crucial role in assisted dying practices, and they are involved in all stages of the process (e.g. in communication and counselling, assessment of eligibility, prescription of lethal drugs, death planning, supervision of the suicide) [6–8]. Nurses often provide consultation and support to healthcare professionals and patients, as well as aftercare for bereaved family members; they sometimes also administer the lethal drugs [9, 10]. Additionally, mental health providers, pharmacists and social workers may be involved in different stages of the process (e.g. consultation, provision of regulatory oversight, drug dosage) and play a critical, but yet under-recognised role in supporting patients’ access to these services and alleviating some of their practical burdens [5, 11–13]. Several studies have also explored the complex role played by family members [14, 15]: On the one hand, family members can act as support figures and caregivers for patients; on the other hand, they can suffer from the patient’s decision and try to convince them not to follow through. A qualitative study from Switzerland indicated that family members might also play a vital role in the organisation of PAS; however, concrete insights into family members’ tasks and perspectives are lacking [16].

In Germany, assisted dying practices have had the characteristics of a taboo and not been openly discussed for a long time. Especially suicide assistance, carried out repeatedly by the same person, was temporarily prohibited from 2015 until 2020. In 2020, the German Federal Constitutional Court overturned the ban on “business-like promotion of suicide” [Geschäftsmäßige Förderung der Selbsttötung] [17]. However, all forms of euthanasia remain prohibited. Since this decision was issued, various draft laws on a new regulation of assisted suicide have been discussed and two cross-party proposals were put to the vote in the German Bundestag in July 2023. However, none of the drafts received the necessary majority, so that assisted suicide remains unregulated in Germany, and discussions about its regulation continue [18, 19].

Meanwhile, official data on the incidence of assisted suicide in Germany are lacking. In 2021, right-to-die organisations provided suicide assistance in almost 350 cases, but other cases of suicide assistance are not systematically documented [20]. There is a lack of scientific evidence about the practice of assisted suicide in general and especially in Germany [21, 22]. Little is known about the role of assisted suicide in the healthcare system, such as palliative care, geriatrics, and general practice. It is unclear where practitioners should turn to for information and advice on the implementation of assisted suicide. This research desideratum is severe, because the lack of clear professional and legal guidelines–and associated lack of knowledge and training–has been identified as a central challenge for practitioners that threatens the practice [4, 5]. In fact, physicians, nurses, and other parties are already providing suicide assistance in Germany, without recourse to scientific or transparent standards, binding guidelines, and quality assurance [17, 23]. Also the special role of right-to-die-organisations in Germany and their involvement in the practice of assisted suicide has been underexplored.

## Materials and methods

### Trial registration

The study was prospectively registered in the German Clinical Trials Register (Deutsches Register Klinischer Studien) (Registration N° DRKS00034574; date of registration: 04 July 2024). The study is searchable under the International Clinical Trials Registry Platform Search Portal of the World Health Organization, under the German Clinical Trials Register number.

### Ethics statement

The study was approved on 22 August 2023 by the Ethics Committee of Hannover Medical School (N° 10196_BO_S_2022). Prior to data collection in all phases of the project, the researchers will provide eligible participants with detailed information about the study type, content, purpose, and duration.

All study participants will be informed in detail, orally and in writing, about the project aims and expected outputs, before confirming their participation in the project. Participation will only be possible when the individual explicitly agrees to participate in the study and signs a written consent form. Each participant will have the right to refuse or discontinue participation at any time without providing any reasons for doing so.

The study results (e.g. transcripts) will be stored pseudonymously on the secure servers of the MHH, in order to ensure personal data protection and prevent the results from being linked to individual participants.

### Study aim

The project “ASEP–Assisted suicide in Germany: Exploring the practice” aims to generate and disseminate scientific knowledge on assisted suicide in Germany, based on the experiences of practitioners and family members.

The three core research questions will be:

1. How do medical and non-medical practitioners and family members experience the current practice of assisted suicide in Germany?
2. Which practical, organisational, ethical, and legal challenges apply to the practice of assisted suicide in Germany?
3. How is assisted suicide linked to the healthcare system in Germany, and where should adjustments to these links be made?

### Design

As Fujioka noted, only few studies (*n* = 5) have addressed the practice of assisted suicide using a qualitative design, and none of these was situated in Germany [4]. Exploratory qualitative approaches can be highly effective at generating insight into understudied fields, and capturing the views and experiences of participants. For this reason, the proposed project will employ an exploratory qualitative study design guided by inductive logic, drawing upon one-time semi-structured interviews and focus groups [24, 25].

**Fig 1:**
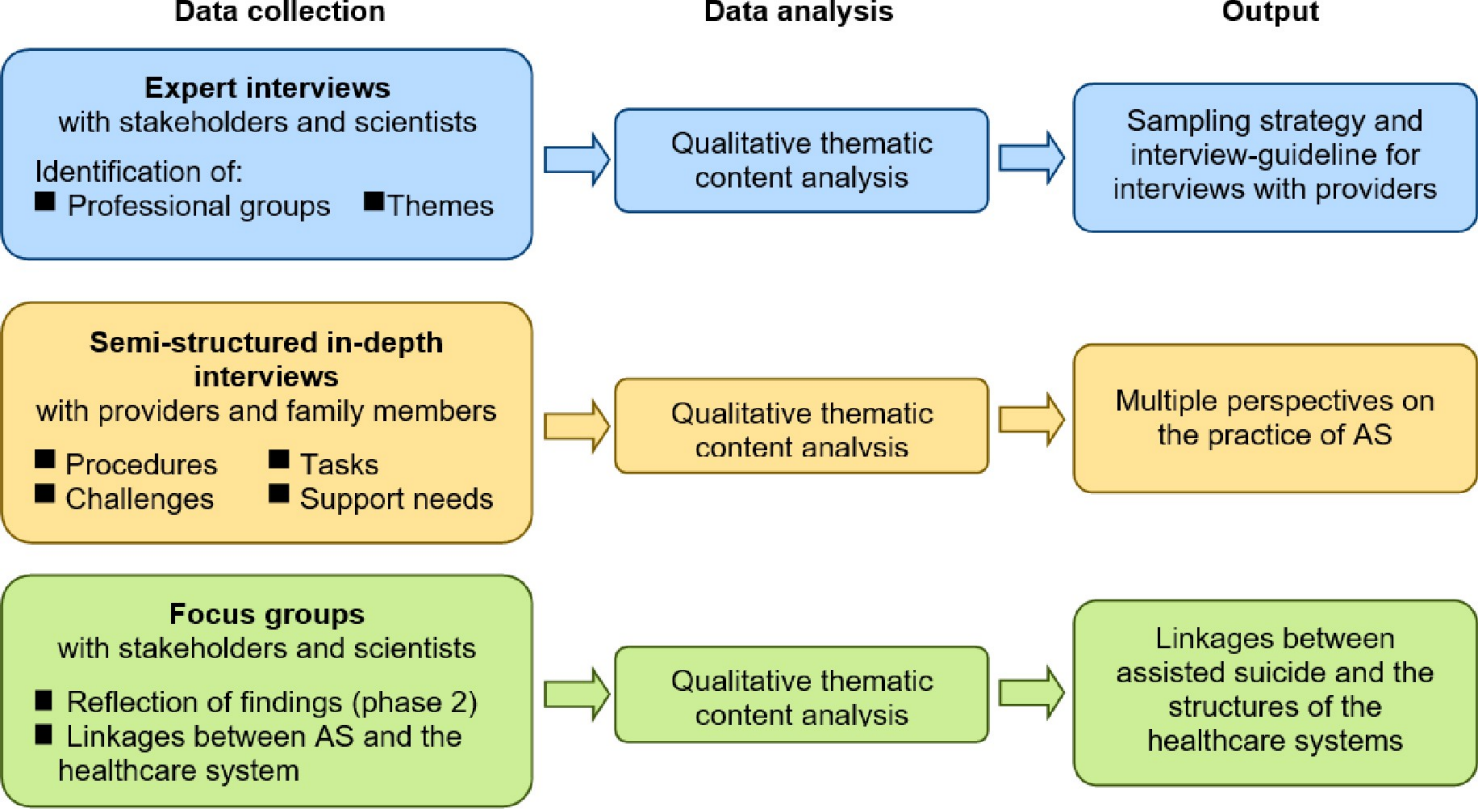
Study design (AS: Assisted suicide).

**Fig 2:**
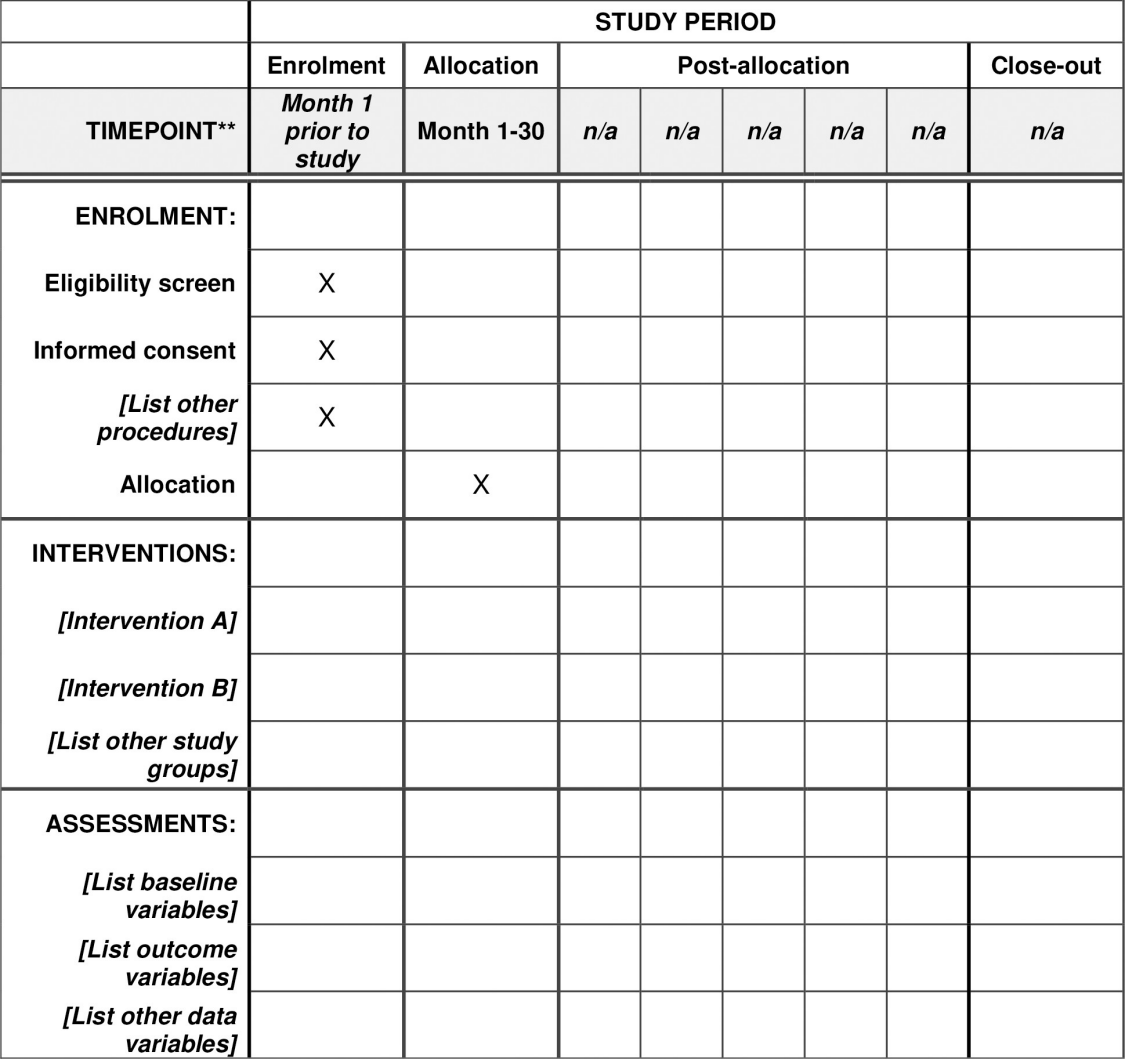
SPIRIT schedule.

The research will be divided into three phases over 2.5 years, focused on: (1) expert interviews (project months 1–6), (2) in-depth interviews with practitioners and family members (project months 7–24) and (3) focus groups (project months 25–30) (see Fig 1). The study is accompanied by a scientific advisory board consisting of experts from the fields of medical law and medical ethics. The involvement of the advisory board is planned in each phase of the project. The study protocol adheres to STROBE guidelines [26]. Data collection can be traced using the spirit schedule (see Fig 2).

#### Work schedule, time frame and milestones

Previous work: To gain an overview of current practices and knowledge about the topic, in November 2021, the applicant conducted *n* = 10 ad-hoc interviews with representatives of right-to-die organisations and physicians associated with the IGPPC. The interviews covered field access, perceived experiences and possible challenges involved in doing research on assisted suicide in Germany. Additionally, an initial screening of the field was performed and a list of experts from science and practice was drawn up. On the basis of the findings, the explorative study design for this project proposal was developed, with the intention of including a broad multiplicity of expert perspectives from science and practice and generating deep insight into the experiences and challenges perceived by practitioners and family members.

#### Phase 1: Expert interviews with stakeholders and scientists (project months 1–6)

In phase 1, expert interviews will be held to identify relevant professional groups and themes for the interview-guideline in phase 2.

The principal investigator will prepare for participant recruitment and begin work on the research infrastructure. At this time, study materials will be prepared, including information for the participants, the data protection concept and a project website.

Building on the previous work and the current state of research, an interview guideline for expert interviews will be developed and experts will be identified.

Experts will be individuals with an overview of and in-depth insight in the practice of assisted suicide in Germany. These may be persons who hold positions of responsibility in relevant associations and institutions (e.g. German Association for Palliative Medicine, German Hospice and Palliative Care Association, German Association for Suicide Prevention, Centre for Health Ethics, German Society for Dying with Dignity) or persons who are identified on the basis of other relevant activities (e.g. scientists with deep insight into the topic, as determined from the literature) from different professions (e.g. medicine, nursing, ethics, law). Patient advocates will also be involved. A list of potential experts has already been drawn up. In total, *n* = 10 national experts will be recruited for the expert interviews. The recruitment period is expected to start on 1 April 2025 and is expected to last three months, until 30 June 2025.

The interview guideline will contain questions about relevant medical and non-medical professional groups and important themes in the German-specific discussion. The results of the interviews will inform the sampling strategy and interview guideline in phase 2. Interviews will be conducted via telephone, via video conference or in person, depending on the preference of each interviewee. Expert interviews will be audio recorded and analysed using thematic content analysis [27].

At the end of phase 1, a half-day online advisory board meeting will be organised to discuss recruitment strategy and themes of the interview guideline. The advisory board will review the accuracy of the overall design, the methodology of the project, public relations and the involvement of interpreters.

#### Phase 2: Semi-structured in-depth interviews with practitioners and family members (project months 7–24)

In phase 2, semi-structured in-depth interviews with practitioners and family members will be held to ascertain their experiences with the current practice of assisted suicide in Germany and perceived organisational and practical challenges.

Drawing on the phase 1 findings, a purposive sampling strategy will be developed for the semi-structured in-depth interviews with practitioners and family members.

An interview guideline will be prepared, tested and revised according to the feedback in phase 1. It is expected that the guideline will include the following domains: 1) the assisted suicide procedure, 2) assisted suicide tasks, 3) assisted suicide challenges and 4) further support needs in the context of assisted suicide. These topics will be modified and added to according to the findings from phase 1. Finally, individual interviews will be conducted with practitioners from different professional groups and family members.

Participants will provide socio-demographic data to inform our analysis of personal characteristics (with respect to, e.g., age, gender, work experience and professional background). Following each qualitative interview, supplementary quantitative data will be collected on the frequency of the interviewee’s participation in assisted suicide and the interviewee’s relationship to the patients in these cases (in terms of duration and the intensity of connection).

The number of interviews will depend on the degree of data saturation, which will be assumed reached when no new codes or themes emerge from the interviews [27, 28]. Research methods guidelines posit that at least 12 interviews are required to achieve data saturation [29]. In the proposed project, a higher number of interviews will be needed, because the project will include participants from different fields and professions (e.g. physicians, nurses, volunteers, family members, lawyers, pharmacists), who have been selected through purposeful sampling [4]. It is expected that the project will administer approximately 30–35 interviews to practitioners and family members, including at least 5 within each sub-group. Interview data will be continuously analysed to determine when data saturation is achieved, at which point data collection will cease [29].

Interviewees are providers in the practice of assisted suicide, ≥ 18 years of age, who were involved in at least one case of assisted suicide in Germany at the time of study participation. Providers may be healthcare professionals from different professions (such as physicians, nurses, psychologists, social work) volunteers of right-to-die organisations, involved lawyers, family members and other players identified in phase 1. Individuals, irrespective of their sex and ethnic background, may participate. Those providers, who fulfil inclusion criteria, will be invited to participate in individual interviews.

**Fig 3:**
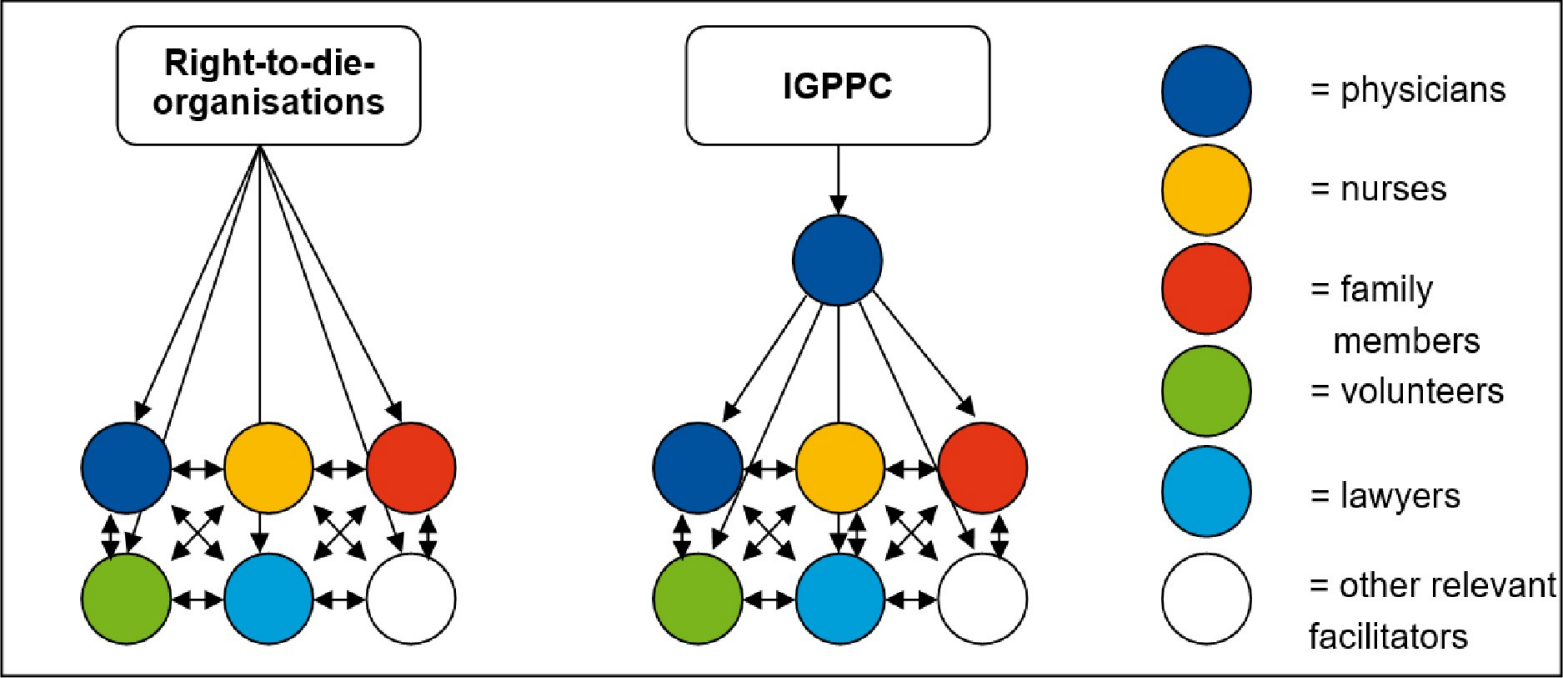
Sampling strategy.

Providers will be recruited via two different ways: 1) request of participating right-to-die organisations on cooperating physicians, nurses, volunteers, lawyers, 2) use of established contacts to general practitioners in Lower-Saxony. Moreover, all interview partners will be asked to suggest further providers and family members, which will be asked for interviews (snowball sampling), according to the sampling strategy (see Fig 3). The recruitment period is expected to start on 1 October 2025 and is expected to last 15 months, until 31 December 2026.

**Fig 4:**
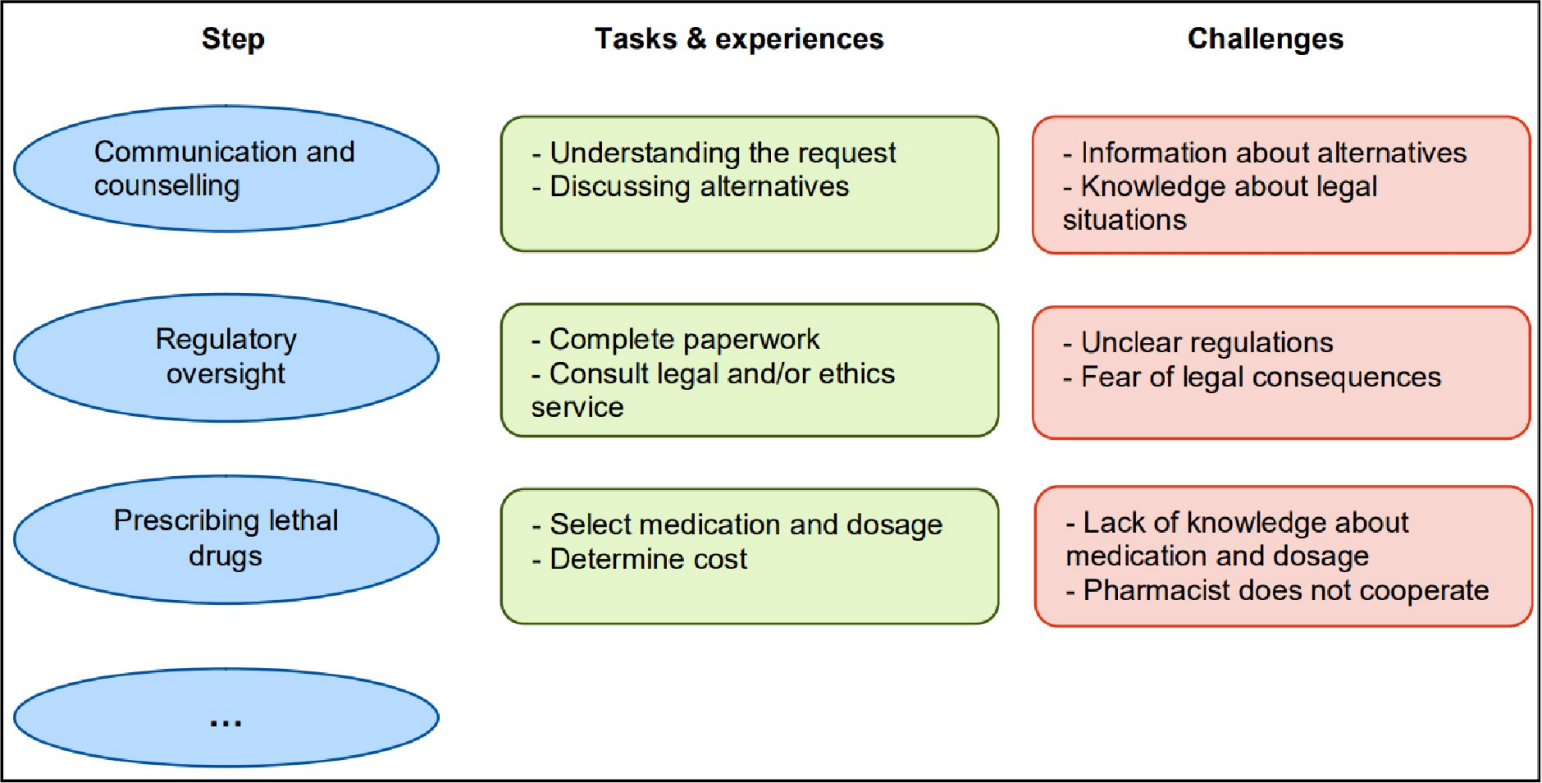
Example of physicians’ tasks and challenges at assisted suicide.

Interviews will be digitally recorded and transcribed verbatim by a research assistant, using the transcription software f4 [30]. Each interview transcript will be analysed by two researchers with the qualitative data analysis software MAXQDA [31], using qualitative content analysis according to Mayring [27]. Data will be analysed via a process of inductive and deductive coding. In the first step of deductive coding, data will be structured according to the main categories, a) procedures, b) tasks, c) challenges and d) support needs. Further, principles of inductive coding are used to develop new codes from the material and to develop codes of existing categories (i.e. category: challenges; codes: organisational challenges, ethical challenges, legal challenges, etc.). In this way, a differentiated code tree will be created that maps all relevant aspects of the practice of assisted suicide (see Fig 4).

Two researchers will independently code interview transcripts. Coding of interviews in categories and codes will take place in an iterative process in which interviews will be coded once conducted. Throughout the coding process, theoretical memos are written to define categories and codes and to trace the process of development of the code tree. Finally, code tree, categories and codes will independently be reviewed by the two researchers, discrepancies will be discussed, and categories and codes will be revised until consent is achieved.

Data bias will be addressed openly during data analysis. The evaluation of qualitative data is always contextually bound. Origin, values, norms, interests, motivation, etc. of the participants are taken into account in the data evaluation. The heterogeneity of the participants and their different points of view, which can be captured by the qualitative interviews used in the project, are relevant for the project, since the aim is to capture subjective perspectives on assisted suicide and to integrate them to paint a multi-perspective picture of the practice of assisted suicide in Germany. Socio-demographic questionnaire data will be entered into IBM SPSS Statistics 26 and analysed descriptively.

In a second online half-day advisory board meeting in project month 10, advisory board members will meet again and discuss preliminary findings emerging from the first three months (7–9) of interviewing. Interviews und participant recruitment will be reflected upon regarding content-related and methodological issues of data collection. Interview guide and recruitment strategy will be modified accordingly if necessary.

#### Phase 3: Focus groups with stakeholders and scientists

In phase 3, focus groups with experts from phase 1 will be conducted to reflect the findings of phase 2 and to discuss linkages between the practice of assisted suicide and the healthcare system.

The code tree and the major findings from the phase 2 interview analysis will be presented and discussed in three half-day focus group discussions with experts. Focus group participants will be comprised of the experts interviewed in phase 1. Additionally, advisory board members from the field of medical ethics and medical law and further experts from the field (who will be identified during the project period) will be involved. As the total number of participants will be greater than 15, three focus groups with heterogeneous participants (*n* = 5–6) will be held, aimed at: (1) discussing the challenges and support needs identified in phase 2, (3) reflecting ethical and legal aspects of the practice of assisted suicide, and (3) reflecting on the linkages between assisted suicide practice and the healthcare system.

The project team will develop a guideline for each focus group, consisting of a) an open brainstorming method (to collect ethical and legal aspects and linkages to the healthcare system) and b) an in-depth workshop phase (to explore and reflect all relevant ethical and legal aspects and linkages to the healthcare system).Focus group results could e.g. encompass information regarding important aspects for professional guidelines, training content for healthcare professionals, access regulations for narcotics, or topics of consultation of suicide prevention. Additionally in the in-depth workshop phase, recommendations will be developed on how these linkages to the healthcare system should be organised (f.e. “healthcare professionals in palliative care should be trained in dealing with dying wishes”). The consensus of all focus group members to the individual recommendations will finally be collected with an online survey using SoSciSurvey [32] and analysed descriptively using Microsoft Excel.

Focus groups will be conducted by the project team and moderated by the team leader, who has significant experience in this role. Focus groups are chosen as a method to sharpen the heterogeneity of the participants’ perspectives and to identify possible lines of conflict.

Focus group data will be audio recorded and analysed using thematic content analysis [27]. Ideas for clarifying the linkages between assisted suicide practice and the healthcare system will be collected and structured thematically according to the target individual (e.g. professional group), institution (i.e. professional association, nursing homes, hospitals), legal/regulatory body or research area. Results will be published in a digital open-access online leaflet. Additionally, a symposium in Hannover (f.e. in cooperation with Zentrum für Gesundheitsethik, Hanns-Lilje Haus, Hannover) for representatives and members of scientific societies (e.g. Deutsche Gesellschaft für Palliativmedizin, Deutscher Hospiz- und PalliativVerband, Gesellschaft für Suizidprävention, etc) and politicians will be organised to provide information and recommendations on possible activities of scientific societies, ethical and organisational aspects of assisted suicide and possible and recommended fields of legal regulation regarding the linkage between assisted suicide and the healthcare system.

### Quality assurance

Torensma et al.’s self-assessment instrument ‘Diversity responsiveness in palliative care projects’ will be administered throughout the project lifetime to ensure diversity responsiveness [33]. During data collection, interviewees’ socio-economic information will be collected and analysed. Participants will also be sampled to achieve variety with respect to key diversity factors, including gender, class, age and cultural background. Furthermore, to encourage insight into the experiences of neglected groups, the interview guideline will feature open questions to allow interviewees space to express experiences and perceived barriers linked to diversity aspects. Finally, during data analysis, the material will be checked for diversity aspects, such as the (potential) role played by cultural background, gender and other diversity factors in patient consultations, assessments of eligibility and confidence in the use of drugs.

## Discussion

### Expected results

The main expected results are: (1) a multi-perspective description of the current practice of assisted suicide in Germany, (2) challenges and support needs of different practitioners and family members regarding the practice of assisted suicide, (3) thematically structured recommendations for the role of assisted suicide in the healthcare system.

The results of the project will support healthcare professionals and experts in the fields of medical ethics and medical law in their work to develop clear regulations and guidelines for assisted suicide.

### Dissemination and implementation

Once data analysis is complete and written up, manuscripts will be published in peer-reviewed, open access journals. Furthermore, the results will be presented at (inter)national conferences in the fields of end-of-life care, health services research and general practice and will be disseminated via the psychosocial care section of the DGP. Data files with no personally identifiable information will be maintained after the study conclusion. In accordance with the APA ethics code section 8.14 “Sharing Research Data for Verification” [34], the project team will disseminate research results in a timely manner and will not withhold unidentifiable data from other professionals seeking to verify the conclusions made by the author(s). Other professionals who wish to investigate new research questions using the data set will need to secure express permission from the IGPPC and the author(s). The IGPPC is well connected to the Medical Chamber of Lower Saxony (Ärztekammer Niedersachsen), the German Association of Palliative Medicine (and their representation in Lower Saxony) and the representation in Lower Saxony of the German Association of General Practitioners; all of these institutional bodies will participate in the public dissemination, transfer and implementation of the project results. Their collaboration has been well established through former joint projects. Educational workshops for in-patient and community palliative care services to communicate the main results of the project will also take place. Recommendations for linkages between assisted suicide practice and the healthcare system will be published in a digital open-access online leaflet, structured thematically according to the different target groups: individuals (e.g. professional group), institutions (i.e. professional association, nursing homes, hospitals), legal/regulatory body or scientific research. Additionally, a symposium for representatives and members of relevant scientific societies (e.g. German Association for Palliative Medicine, **German Hospice** and **Palliative Association**, German **Association** for Suicide Prevention, German Association for General Practice and Family Medicine) and politicians will be organised to provide information on possible activities of scientific societies, ethical and organisational aspects of assisted suicide and possible fields of legal regulation regarding the linkage between assisted suicide and the healthcare system.

Finally, a report on the main results will be published on the IGPPC website and communicated at relevant working groups and to practitioners.

## Supporting information

S1 Checklist STROBE statement—Checklist of items that should be included in reports of observational studies. (PDF)

S2 Checklist Human participants research checklist. (PDF)
