# Supplementary material for: Suicide assistance in Germany: A protocol for a multi-perspective qualitative study to explore the current practice
Source: PLoS One. 2024 Dec 13;19(12):e0311880. doi: 10.1371/journal.pone.0311880 (PMC11642949; doi:10.1371/journal.pone.0311880)
Supplement: Attachment — Submitted filename: Response to the editors.docx [file pone.0311880.s003.docx]

MHH

Editors in Chief

*PLOS ONE*

Institut für Allgemeinmedizin und Palliativmedizin

Direktor: Prof. Dr. Nils Schneider, MPH

Wissenschaftlicher Mitarbeiter

Dr. Sven Schwabe

Telefon:	0511 532-8599

Fax: 0511 532-4176

Schwabe.sven@mh-hannover.de

Carl-Neuberg-Straße 1

30625 Hannover

Telefon: 0511 532-0

www.mhh.de

17. September 2024

Dear Editors,

thank you for giving us the opportunity to submit a revision of our manuscript “Exploring the practice of suicide assistance in Germany: A protocol for a multi-perspective qualitative study” (PONE-D-24-36986).

We would like to thank the editors for their comments and valuable suggestions. We have considered them carefully in the revision of the manuscript:

| **Comment** | **Response** |
| --- | --- |
| 1. We would require your ethics committee to formally confirm that ethical approval was not needed in this case for the ad-hoc interviews conducted in November 2021. | Confirmation of the ethic committee is attached. |
| 2. Please provide additional details regarding participant consent. In the ethics statement in the Methods and online submission information, please ensure that you have specified (1) whether consent was informed and (2) what type you obtained (for instance, written or verbal, and if verbal, how it was documented and witnessed). If your study included minors, state whether you obtained consent from parents or guardians. If the need for consent was waived by the ethics committee, please include this information. If you are reporting a retrospective study of medical records or archived samples, please ensure that you have discussed whether all data were fully anonymized before you accessed them and/or whether the IRB or ethics committee waived the requirement for informed consent. If patients provided informed written consent to have data from their medical records used in research, please include this information | Additional details regarding participant consent are provided in the ethics statement and in the online submission information. |
| 3. Please report in the Methods section the day, month and year of the start and tentative end of the recruitment period for this study. If the recruitment periods differ for each phase of the study please include the relevant dates for each phase of the study. Please note that if this information is not included when your manuscript is resubmitted, it may be rejected. | Recruitment periods have been added in phase 1 and 2.  For phase 3, it has already been described that the participants are identical to those from phase 1 and that possible additional participants will be identified during the project period. |

We graciously thank you for your time in evaluating the revised version of our manuscript.

Yours sincerely,

Sven Schwabe
